# Dynamic development of psychological resources among parents and primary caregivers in the context of home-kindergarten co-education: bidirectional associations and mediating pathways in a Chinese sample

**DOI:** 10.3389/fpsyg.2026.1795476

**Published:** 2026-08-04

**Authors:** Shenghua Gong

**Affiliations:** School of Preschool Education, Qilu Normal University, Jinan, China

**Keywords:** home–kindergarten co-education, life satisfaction, longitudinal mediation, parents and primary caregivers, personal growth initiative, psychological resilience

## Abstract

**Introduction:**

Against the background of China’s policy emphasis on home–kindergarten co-education, this study examined the developmental patterns and interrelations of three psychological resources among parents and primary caregivers of preschool children: personal growth initiative, psychological resilience, and life satisfaction.

**Methods:**

A mixed design combined a baseline cross-sectional survey with a three-wave longitudinal follow-up conducted at 3-month intervals over 6 months. Participants were recruited through convenience sampling from eight kindergartens (five public, three private) in five cities across three Chinese provinces. The baseline sample comprised 856 caregivers (62.03% female; mean age = 36.82 years, SD = 7.45; range 25–55 years; 50.24% holding a junior college degree or above; 87.38% married), of whom 689 completed all three waves (attrition = 19.51%). Personal growth initiative, psychological resilience, and life satisfaction were assessed with the Personal Growth Initiative Scale-II, the Connor–Davidson Resilience Scale, and the Satisfaction with Life Scale, respectively, with demographic and socioeconomic characteristics included as covariates. Data were analyzed using Pearson correlations, PROCESS-based mediation analysis with 5,000 bootstrap resamples, latent growth models, cross-lagged panel models (CLPM), random-intercept cross-lagged panel models (RI-CLPM), and longitudinal mediation analysis.

**Results:**

At baseline, the three resources were positively correlated, and psychological resilience partially mediated the association between personal growth initiative and life satisfaction. Latent growth models indicated significant increases in personal growth initiative and psychological resilience, whereas life satisfaction remained relatively stable. CLPM analyses suggested modest bidirectional associations between personal growth initiative and resilience, which were partly retained at the within-person level in the RI-CLPM, although later-wave effects were weaker. T2 psychological resilience mediated the association between T1 personal growth initiative and T3 life satisfaction. Female participants, those aged 36–45 years, and those with higher educational attainment generally reported stronger psychological resources.

**Conclusion:**

Psychological resilience may serve as an important bridge between proactive growth motivation and wellbeing in home–kindergarten partnership contexts. The findings provide implications for differentiated psychological support programs for families of preschool children.

## 1 Introduction

Home-kindergarten co-education has become an important policy and practice framework for the high-quality development of preschool education in China. It emphasizes the shared responsibility of families, kindergartens, and the wider community in supporting young children’s development. Prior research has repeatedly shown that family engagement in early childhood education is associated with children’s cognitive, social, and emotional outcomes (Epstein, 2019; Fan and Chen, 2001; Ma et al., 2016). In China, policy documents such as the Family Education Promotion Law of the People’s Republic of China and the Kindergarten Education Guidelines (Trial) have further highlighted the need to establish collaborative child-rearing communities across homes, kindergartens, and society (Ministry of Education of the People’s Republic of China, 2001; National People’s Congress Standing Committee, 2021; Ministry of Education of the People’s Republic of China, National Development and Reform Commission, Ministry of Public Security, Ministry of Finance, Ministry of Human Resources and Social Security, Ministry of Natural Resources, et al., 2021).

Although the structural and behavioral dimensions of family-kindergarten cooperation have received increasing attention (Fantuzzo et al., 2004; Hoover-Dempsey and Sandler, 1997), less is known about the psychological resources that sustain adults’ participation in such partnerships over time. The present study focuses on three psychological constructs that are especially relevant for parents and primary caregivers in the home-kindergarten co-education context: personal growth initiative, psychological resilience, and life satisfaction. Personal growth initiative reflects adults’ proactive motivation to improve themselves and make use of resources. Psychological resilience reflects their capacity to adapt to parenting stress and recover from difficulties. Life satisfaction captures their overall evaluation of life quality and family wellbeing.

The study uses the expression “parents and primary caregivers” to refer to adults who assume primary responsibility for the daily care and educational involvement of preschool children. This includes biological parents and, where applicable, grandparents or other family members who serve as primary caregivers. This broader term is important in the Chinese preschool context, where delayed childbearing, second- or third-child families, reconstituted families, and intergenerational caregiving have produced a wider age span among adults involved in children’s education (Chen et al., 2020; National Bureau of Statistics of China, 2021).

Theoretically, this study integrates self-determination theory, conservation of resources theory, and ecological systems theory. Self-determination theory explains why autonomous motivation may support sustained engagement in parenting and co-education activities (Deci and Ryan, 1985, 2000). Conservation of resources theory explains how psychological resources accumulate, buffer stress, and may form positive resource spirals (Hobfoll, 2001). Ecological systems theory clarifies why home-kindergarten partnerships can be understood as a mesosystem context in which families and institutions jointly shape adult and child development (Bronfenbrenner, 1979). Together, these frameworks support a context–motivation–resources–outcomes logic: home-kindergarten co-education provides a contextual background; personal growth initiative represents a motivational resource; psychological resilience functions as an adaptive resource; and life satisfaction represents a wellbeing outcome.

### 1.1 Home-kindergarten co-education as the context of the study

Home-kindergarten co-education refers to a policy-driven partnership model in China that emphasizes collaboration among families, kindergartens, and society. Internationally, it is related to concepts such as family-school partnerships, home-school collaboration, and co-parenting alliances between families and educational institutions (Christenson and Reschly, 2010; Epstein, 2019; McWayne et al., 2004). However, the Chinese model is distinctive because it is embedded in a top-down policy system and is often implemented through parent committees, home visits, open classroom days, family education workshops, and digital communication platforms.

At the policy level, China has developed a series of legal and administrative documents supporting home-kindergarten-society collaboration. These include the Law of the People’s Republic of China on the Protection of Minors, China Education Modernization 2035, and more recent documents on collaborative child-rearing mechanisms (Central Committee of the Communist Party of China and State Council, 2019; Ministry of Education of the People’s Republic of China, Publicity Department of the CPC Central Committee, Office of the Central Cyberspace Affairs Commission, Office of the Central Guidance Commission on Building Spiritual Civilization, Ministry of Public Security, Ministry of Civil Affairs, et al., 2022; Ministry of Education of the People’s Republic of China, Publicity Department of the CPC Central Committee, Office of the Central Cyberspace Affairs Commission, Ministry of Science and Technology, Ministry of Public Security, Ministry of Civil Affairs, et al., 2024; National People’s Congress Standing Committee, 2020). These policies provide the institutional background for studying the psychological resources of adults who participate in early childhood education.

In practice, however, home-kindergarten co-education still faces several limitations. Communication often remains asymmetrical, with kindergartens sending information to families rather than engaging parents and caregivers as substantive partners. Participation may be concentrated in event attendance rather than decision-making or sustained learning. Moreover, psychological support for parents and caregivers is rarely treated as a core component of co-education (Li and Wang, 2022; Peng and Wang, 2024). A meta-analysis by Tyler et al. (2020) confirmed that family-school partnership interventions yielded positive effects on children’s academic and social-emotional outcomes, yet the mechanisms through which parental psychological traits mediate these effects remain underexplored. Therefore, examining psychological resources in this context can help clarify how families may be better supported beyond structural participation alone.

### 1.2 Personal growth initiative among parents and primary caregivers

Personal growth initiative is a positive psychological construct (Seligman and Csikszentmihalyi, 2000) referring to individuals’ active and intentional involvement in changing and developing themselves (Robitschek, 1998; Robitschek et al., 2012). It includes readiness for change, planfulness, using resources, and intentional behavior. In the context of preschool education, adults with higher personal growth initiative may be more willing to seek parenting information, reflect on their own parenting practices, communicate with teachers, and participate in family-kindergarten activities.

The construct is theoretically connected to self-determination theory and social cognitive theory (Bandura, 1986; Deci and Ryan, 1985; Zimmerman, 1989). Existing research has linked personal growth initiative to psychological wellbeing, lower distress, and adaptive coping (Robitschek and Keyes, 2009; Shorey et al., 2015). In educational contexts, individuals with higher personal growth initiative are more likely to seek out learning resources, engage in reflective practices, and participate actively in institutional programs (Stevic and Ward, 2008; Sun et al., 2014). Longitudinal evidence suggests that personal growth initiative is not merely a stable trait but exhibits meaningful within-person variability, responding to environmental supports and life transitions (Robitschek et al., 2012). This dynamic quality makes it particularly relevant in the context of home-kindergarten co-education, where policy-driven participation opportunities may activate or sustain caregivers’ growth motivation.

However, most studies on personal growth initiative have focused on college students, adolescents, or clinical populations. Research on general parents and caregivers of preschool children remains limited, particularly in non-Western co-education contexts (Yang and Chang, 2014; Fan et al., 2023). Furthermore, no study to date has examined the bidirectional temporal dynamics between personal growth initiative and other psychological resources in a home-kindergarten co-education sample, leaving a clear gap that the present study aims to address.

### 1.3 Psychological resilience among parents and primary caregivers

Psychological resilience refers to the capacity to maintain or regain adaptive functioning when facing stress or adversity (Lazarus and Folkman, 1984; Luthar et al., 2000; Masten, 2001, 2007; Rutter, 1987). For adults raising preschool children, resilience is relevant because early childhood parenting often involves emotional labor, time pressure, educational anxiety, and family-work conflicts. Resilience may help caregivers cope with setbacks in parenting and maintain constructive involvement with kindergartens.

A substantial body of research has demonstrated that parental resilience is positively associated with adaptive parenting behaviors, reduced parenting stress, and better child outcomes (Hu et al., 2015; Rakap and Vural-Batik, 2024; Zhu et al., 2024). Parental resilience is not only an individual characteristic; it is also shaped by family, community, and institutional resources (Masten and Monn, 2015; Walsh, 2016). In the home-kindergarten context, teacher consultations, parenting workshops, peer support, and community services may function as external protective factors that bolster parental resilience (García et al., 2023).

Despite these advances, several gaps persist. First, most empirical studies on parental resilience have used cross-sectional designs, limiting understanding of how resilience develops over time (Hu et al., 2015). Second, research has predominantly focused on families experiencing special adversity or parents of children with special needs (Rakap and Vural-Batik, 2024), with general populations of preschool families underrepresented. Third, the reciprocal relationship between resilience and other psychological resources—particularly growth initiative—has rarely been tested longitudinally, leaving unresolved whether resilience is primarily an outcome of proactive growth or also a driver of it.

### 1.4 Life satisfaction among parents and primary caregivers

Life satisfaction is the cognitive component of subjective wellbeing and reflects individuals’ global evaluation of their life conditions (Diener et al., 1999; Michalos, 1985; Pavot and Diener, 2008). For parents and primary caregivers of preschool children, life satisfaction may be influenced by parenting stress, family functioning, socioeconomic resources, and the quality of home-kindergarten communication (Matalon et al., 2022). Adults with higher life satisfaction may also display more emotional availability and constructive educational involvement (Zakirova-Engstrand and Wilder, 2024).

At the same time, life satisfaction tends to be relatively stable over short periods because of hedonic adaptation and baseline tendencies in subjective wellbeing (Brickman and Campbell, 1971; Lucas, 2007). This suggests that life satisfaction may not change as quickly as more malleable psychological resources such as personal growth initiative or resilience. Understanding this difference in temporal dynamics is important for designing and evaluating parent-support interventions.

### 1.5 Relationships among the three constructs

The three constructs may be linked through a motivation–resources–outcomes pathway. Personal growth initiative represents a proactive motivational tendency. Individuals with higher growth initiative are more likely to use available resources, engage in reflective learning, and seek social support (Robitschek and Keyes, 2009; Sun et al., 2014). These behaviors may contribute to the development of psychological resilience. Resilience, in turn, may help adults appraise parenting and family challenges more constructively, which may be associated with higher life satisfaction (Hu et al., 2015; García et al., 2023).

Conservation of resources theory further suggests that these relationships may be reciprocal. Resource gains can reinforce motivation for additional investment, producing a resource spiral (Hobfoll, 2001). Thus, personal growth initiative may support resilience, while resilience may also make continued personal growth more likely. Preliminary longitudinal evidence from non-parental populations supports such reciprocal dynamics (Smith et al., 2022). However, previous research has rarely examined these reciprocal pathways longitudinally among preschool parents and caregivers, and no study has simultaneously tested bidirectional predictive relationships and mediating pathways among these three constructs within a home-kindergarten co-education context.

### 1.6 Integrative theoretical framework and research questions

**FIGURE 1:**
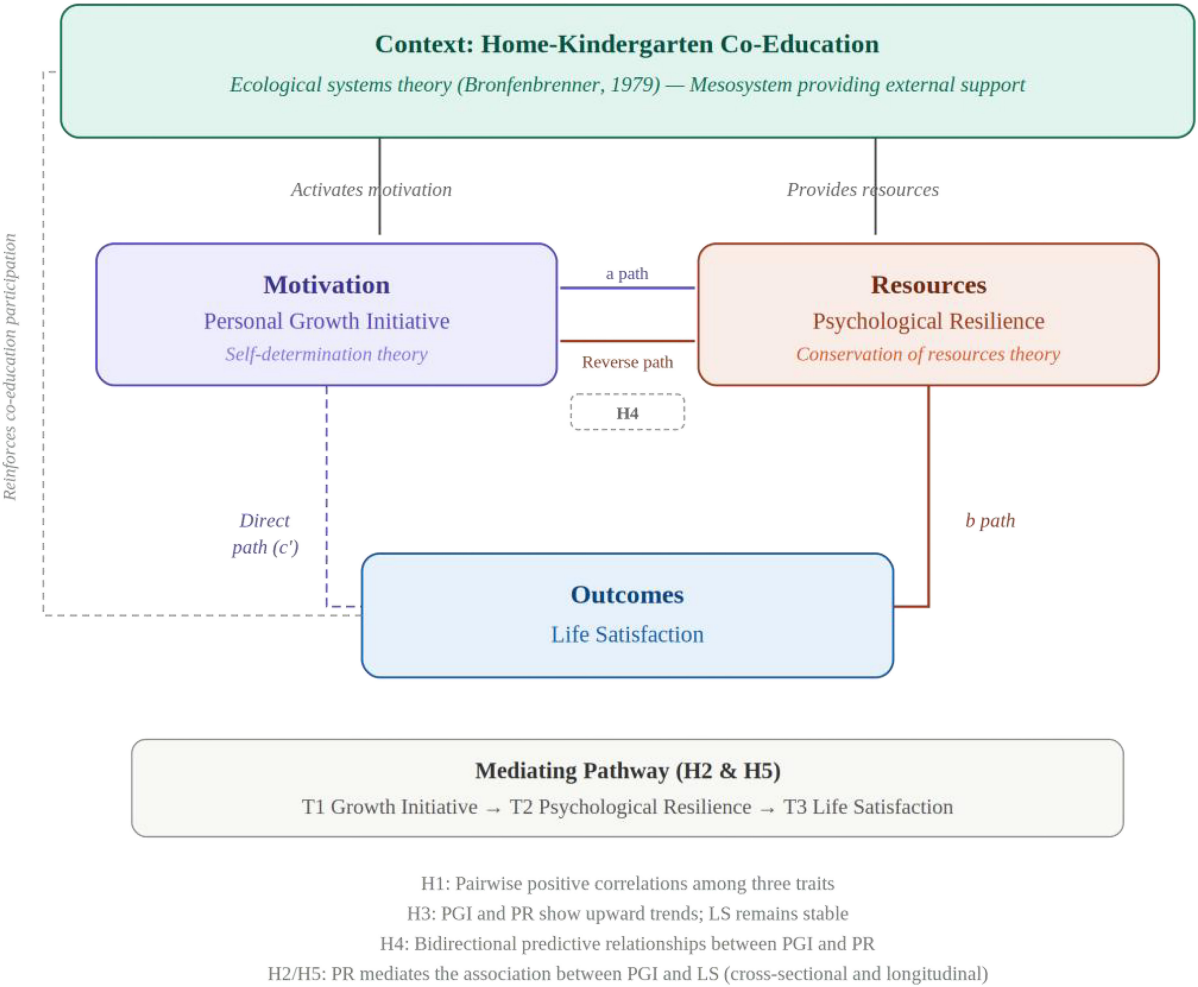
Integrative theoretical framework of the context–motivation–resources–outcomes pathway.

This study proposes a context–motivation–resources–outcomes framework. Home-kindergarten co-education provides the contextual background. Personal growth initiative serves as the motivational component. Psychological resilience represents an adaptive resource that may both result from and reinforce proactive growth. Life satisfaction represents a broader wellbeing outcome. Figure 1 presents the conceptual framework.

Based on this framework, the study addressed five hypotheses:

*H1:* Personal growth initiative, psychological resilience, and life satisfaction are positively associated. Rationale: Prior cross-sectional evidence has demonstrated positive associations among proactive personality traits, adaptive coping resources, and subjective wellbeing indicators (Hu et al., 2015; Robitschek and Keyes, 2009). Self-determination theory predicts that autonomous motivation facilitates psychological resource acquisition, which in turn enhances life evaluation (Deci and Ryan, 2000).

*H2*: Psychological resilience statistically mediates the association between personal growth initiative and life satisfaction. Rationale: Conservation of resources theory posits that resource accumulation serves as an intermediary process linking motivational investment to wellbeing outcomes (Hobfoll, 2001). Cross-sectional studies on parent populations have provided preliminary support for this mediating pathway (García et al., 2023; Hu et al., 2015).

*H3*: Over 6 months, personal growth initiative and psychological resilience show upward linear trends, whereas life satisfaction remains relatively stable. Rationale: Continued participation in home-kindergarten co-education activities is expected to provide ongoing opportunities for resource development, consistent with conservation of resources theory’s resource gain spirals (Hobfoll, 2001). The predicted stability of life satisfaction is consistent with hedonic adaptation theory (Brickman and Campbell, 1971; Lucas, 2007).

*H4*: Personal growth initiative and psychological resilience show reciprocal longitudinal associations. Rationale: Conservation of resources theory’s resource spiral hypothesis predicts that resource gains reinforce the motivation to invest further, suggesting reciprocal temporal associations (Hobfoll, 2001). Preliminary longitudinal evidence supports such reciprocal dynamics in parental populations (Smith et al., 2022).

*H5*: T2 psychological resilience mediates the association between T1 personal growth initiative and T3 life satisfaction, after controlling for baseline levels and demographic covariates. Rationale: The temporal sequencing of the motivation → resources → outcomes chain predicts that T1 growth initiative relates to T3 life satisfaction through T2 resilience accumulation (Hobfoll, 2001; Hayes, 2018).

## 2 Materials and methods

### 2.1 Research design

This study adopted a mixed design combining a baseline cross-sectional survey and a three-wave longitudinal follow-up. The cross-sectional component was used to describe the status of the three psychological resources, examine their associations, and test a mediation model. The longitudinal component was used to examine developmental trajectories, reciprocal associations, and a longitudinal mediation pathway over 6 months.

### 2.2 Participants and sampling

Participants were recruited through convenience sampling from eight kindergartens in five Chinese cities across three provinces. The kindergarten sample included five public and three private kindergartens, and the cities included one first-tier city, two new first-tier cities, and two prefecture-level cities. The target participants were parents and primary caregivers who were primarily responsible for preschool children’s daily care and educational involvement.

A priori power analysis was conducted using G*Power 3.1 (Faul et al., 2009). For the cross-sectional mediation model, assuming a medium effect size, α = 0.05, and power = 0.95, the minimum required sample size was 146. For the longitudinal structural equation models, the sample size also exceeded commonly cited recommendations of 10–20 participants per estimated parameter (Kline, 2016). Thus, both the cross-sectional and longitudinal samples were adequate for the planned analyses.

#### 2.2.1 Baseline cross-sectional sample

A total of 880 questionnaires were distributed at baseline. After 867 were returned and 11 invalid questionnaires were excluded, 856 valid questionnaires remained, yielding an effective recovery rate of 97.27%. The mean age of participants was 36.82 years (SD = 7.45). The sample included 325 males (37.97%) and 531 females (62.03%). In terms of age, 368 participants were aged 25–35 years (43.00%), 296 were aged 36–45 years (35.58%), and 192 were aged 46–55 years (22.43%). The older group included older parents of second or third children, primary caregivers in reconstituted families, and grandparents who assumed primary caregiving responsibilities.

Regarding education, 128 participants had junior high school education or below (14.95%), 298 had high school or vocational school education (34.81%), 312 had junior college or bachelor’s degrees (36.45%), and 118 had master’s degrees or above (13.79%). Regarding marital status, 748 were married (87.38%), 62 were divorced (7.24%), 18 were widowed (2.10%), and 28 were single (3.27%). Regarding number of children, 386 had one child (45.09%), 378 had two children (44.16%), and 92 had three or more children (10.75%). Annual household income was below ¥50,000 for 108 participants (12.62%), ¥50,000–100,000 for 246 (28.74%), ¥100,000–200,000 for 312 (36.45%), ¥200,000–300,000 for 128 (14.95%), and above ¥300,000 for 62 (7.24%).

#### 2.2.2 Longitudinal sample

Among the 856 participants who completed the baseline survey, 689 completed the three-wave follow-up and were included in the longitudinal analyses. The three waves were conducted in September 2024 (T1), December 2024 (T2), and March 2025 (T3), with an interval of approximately 3 months between adjacent waves. A total of 167 baseline participants did not complete one or more follow-up assessments and were treated as attrited cases. The attrition rate was 19.51%.

Independent-samples *t*-tests indicated no significant differences between retained and attrited participants in baseline personal growth initiative, psychological resilience, life satisfaction, or major demographic characteristics. These results suggested no evidence of systematic attrition. Item-level missing values were examined using Little’s MCAR test (Little, 1988). Results indicated that missingness was consistent with the missing completely at random assumption, χ^2^(246) = 261.34, *p* = 0.23. Full information maximum likelihood estimation was used in Mplus analyses to handle missing data (Enders, 2010).

### 2.3 Measures

#### 2.3.1 Personal growth initiative

Personal growth initiative was measured using the Personal Growth Initiative Scale-II developed by Robitschek et al. (2012) and adapted for Chinese samples by Yang and Chang (2014). The scale includes 16 items covering readiness for change, planfulness, using resources, and intentional behavior. Items were rated on a 6-point Likert scale ranging from 0 (completely disagree) to 5 (completely agree). In the present study, Cronbach’s α was 0.87. Confirmatory factor analysis supported the four-factor structure: χ^2^(98) = 286.45, CFI = 0.95, TLI = 0.94, RMSEA = 0.048, SRMR = 0.042. Standardized factor loadings ranged from 0.58 to 0.82. Average variance extracted for each dimension exceeded 0.50 (readiness for change = 0.52, planfulness = 0.55, using resources = 0.53, intentional behavior = 0.54), supporting convergent validity. Discriminant validity was confirmed as the square root of each dimension’s AVE exceeded its correlations with other dimensions.

#### 2.3.2 Psychological resilience

Psychological resilience was measured using the Chinese version of the Connor-Davidson Resilience Scale (Connor and Davidson, 2003; Yu and Zhang, 2007), one of the most widely used resilience measures alongside the Resilience Scale (Wagnild and Young, 1993). The scale contains 25 items and covers tenacity, strength, and optimism. Items were rated on a 5-point Likert scale ranging from 0 (never) to 4 (almost always). Cronbach’s α was 0.91. Confirmatory factor analysis supported the three-factor structure: χ^2^(272) = 624.38, CFI = 0.94, TLI = 0.93, RMSEA = 0.039, SRMR = 0.038. Standardized factor loadings ranged from 0.55 to 0.85. AVE values exceeded 0.50 for all dimensions (tenacity = 0.53, strength = 0.56, optimism = 0.54).

#### 2.3.3 Life satisfaction

Life satisfaction was measured using the Chinese version of the Satisfaction with Life Scale (Diener et al., 1985; Pavot and Diener, 1993; Xiong and Xu, 2009). The scale includes five items rated on a 7-point Likert scale ranging from 1 (strongly disagree) to 7 (strongly agree). Cronbach’s α was 0.84. Confirmatory factor analysis supported the one-factor structure: χ^2^(5) = 18.62, CFI = 0.99, TLI = 0.98, RMSEA = 0.056, SRMR = 0.021. Standardized factor loadings ranged from 0.64 to 0.83.

#### 2.3.4 Covariates

The main covariates included gender, age, education level, marital status, number of children, and socioeconomic status. Socioeconomic status was represented by annual household income on a 5-point scale. Parenting stress was also assessed using the short form of the Parenting Stress Index (Abidin, 1995). Primary models controlled for demographic and socioeconomic covariates. Additional robustness checks including parenting stress yielded substantively similar results, suggesting that the main associations were not solely attributable to stress-related confounding.

### 2.4 Procedure

Data collection was organized by uniformly trained kindergarten staff. Baseline questionnaires were administered through a combination of household distribution and centralized kindergarten administration. Participants completed the questionnaires anonymously. For longitudinal tracking, participants generated unique 10-character alphanumeric codes at T1, which were used to match responses across T2 and T3 while protecting anonymity. Data collection procedures were standardized across kindergartens.

### 2.5 Data analysis

Data were entered using EpiData 3.1 with double entry and cross-verification. SPSS 26.0 was used for data cleaning, descriptive statistics, Pearson correlations, reliability analysis, demographic difference tests, and cross-sectional mediation analysis. Hayes’ PROCESS macro (Model 4) was used to test the cross-sectional mediation model with 5,000 bootstrap resamples.

Mplus 8.3 was used for longitudinal analyses. Latent growth models examined developmental trajectories across T1, T2, and T3. Cross-lagged panel models examined reciprocal associations between personal growth initiative and psychological resilience while controlling for autoregressive effects. Because traditional cross-lagged panel models can conflate between-person and within-person effects (Hamaker et al., 2015), supplementary random-intercept cross-lagged panel models were also estimated. With only three measurement waves, the RI-CLPM’s additional parameters may introduce estimation instability (Berry and Willoughby, 2017); results from both models are therefore reported for transparency. Longitudinal mediation analysis tested whether T2 psychological resilience mediated the association between T1 personal growth initiative and T3 life satisfaction while controlling for baseline psychological resilience, baseline life satisfaction, and demographic covariates.

Measurement invariance across the three waves was tested for all three scales. Configural, metric, and scalar invariance were evaluated using changes in CFI and RMSEA. The criteria of ΔCFI ≤ 0.01 and ΔRMSEA ≤ 0.015 were used to judge invariance. Results supported cross-time measurement invariance for all three constructs.

## 3 Results

### 3.1 Common method bias

Because the main variables were measured through self-report questionnaires, common method bias was assessed. Harman’s single-factor test (Zhou and Long, 2004) showed that the first unrotated factor explained 27.86% of the variance at baseline, below the commonly used 40% threshold. In addition, a confirmatory factor analysis model including an unmeasured latent method factor was compared with the baseline measurement model (Podsakoff et al., 2003; Williams et al., 2010). Adding the method factor did not substantially improve model fit (ΔCFI = 0.003, ΔRMSEA = 0.002), and the average method factor loading was 0.08, accounting for approximately 3.6% of item variance. These results suggested that common method bias was unlikely to fully account for the observed associations.

### 3.2 Descriptive statistics and correlations at baseline

Table 1 presents the means, standard deviations, and Pearson correlations among the three focal variables at baseline. Personal growth initiative, psychological resilience, and life satisfaction were all at moderately high levels and were significantly positively correlated with one another (*r* = 0.38–0.46, *p* < 0.001). These results supported H1 and provided the basis for subsequent mediation analyses.

**TABLE 1:** Means, standard deviations, and baseline correlations (*n* = 856).

Variable	*M*	SD	1	2	3
1. Personal growth initiative	3.52	0.61	1	1	1
2. Psychological resilience	3.81	0.64	0.41***		
3. Life satisfaction	5.05	0.91	0.38***	0.46***	

***p < 0.001.

### 3.3 Cross-sectional mediation analysis

After controlling for gender, age, education, marital status, number of children, and socioeconomic status, personal growth initiative positively predicted psychological resilience and life satisfaction (Table 2). Psychological resilience also positively predicted life satisfaction. Bootstrap analysis showed that the indirect effect of personal growth initiative on life satisfaction through psychological resilience was significant, with a 95% confidence interval that did not include zero (Table 3). The mediation was partial rather than complete, indicating that personal growth initiative remained directly associated with life satisfaction after accounting for psychological resilience. These results supported H2.

**TABLE 2:** Cross-sectional mediation model for psychological resilience.

Path	β	*t*	*p*
PGI → PR	0.42	11.28	<0.001
PGI → LS (total)	0.47	15.75	<0.001
PGI → LS (direct)	0.29	9.74	<0.001
PR → LS	0.43	12.93	<0.001

PGI, personal growth initiative; PR, psychological resilience; LS, life satisfaction. Models controlled for gender, age, education, marital status, number of children, and SES.

**TABLE 3:** Bootstrap test of the indirect effect of psychological resilience.

Effect	Estimate	SE	95% LL	95% UL	Proportion
Total	0.47	0.03	0.41	0.53	100%
Direct	0.29	0.03	0.23	0.35	61.70%
Indirect	0.18	0.02	0.14	0.23	38.30%

Bootstrap resamples = 5,000. LL, lower limit; UL, upper limit.

### 3.4 Measurement invariance across three waves

Before testing longitudinal models, measurement invariance was evaluated across T1, T2, and T3. Configural, metric, and scalar invariance were supported for all three scales (Table 4), with ΔCFI values not exceeding 0.01 and ΔRMSEA values not exceeding 0.015. These findings indicated that the constructs were measured comparably across time, supporting the validity of longitudinal comparisons.

**TABLE 4:** Measurement invariance testing across three waves.

Scale	Model	χ ^2^(df)	CFI	RMSEA	Δ CFI	Δ RMSEA
PGIS-II	Config.	582.45 (294)	0.952	0.046	–	–
	Metric	618.72 (318)	0.949	0.045	0.003	0.001
	Scalar	662.38 (342)	0.945	0.045	0.004	0.000
CD-RISC	Config.	1486.52 (816)	0.943	0.042	–	–
	Metric	1548.36 (860)	0.940	0.042	0.003	0.000
	Scalar	1628.74 (904)	0.936	0.043	0.004	0.001
SWLS	Config.	38.24 (15)	0.988	0.048	–	–
	Metric	46.82 (23)	0.986	0.044	0.002	0.004
	Scalar	58.36 (31)	0.982	0.043	0.004	0.001

ΔCFI ≤ 0.01 and ΔRMSEA ≤ 0.015 supported invariance across time.

### 3.5 Demographic differences

Table 5 presents baseline demographic differences in the three focal variables. Female participants reported higher personal growth initiative and psychological resilience than male participants, whereas the gender difference in life satisfaction was not significant. Participants aged 36–45 reported higher psychological resilience than other age groups, and older participants showed relatively high life satisfaction. Higher educational attainment was associated with higher levels of all three variables.

**TABLE 5:** Baseline demographic differences in the focal variables.

Variable	Trait	Group 1	Group 2	Group 3	Group 4	Stat
Gender	PGI	M: 3.48 ± 0.64	F: 3.55 ± 0.58			*t* = -2.28*
	PR	M: 3.64 ± 0.67	F: 3.77 ± 0.64			*t* = -2.56**
	LS	M: 5.05 ± 0.89	F: 5.10 ± 0.86			*t* = -0.68
Age	PGI	25–35: 3.53	36–45: 3.58	46–55: 3.54		*F* = 0.72
	PR	25–35: 3.65	36–45: 3.82	46–55: 3.68		*F* = 4.35**
	LS	25–35: 4.98	36–45: 5.12	46–55: 5.18		*F* = 3.25*
Edu.	PGI	≤ JH: 3.42	HS: 3.52	BA: 3.59	MA+: 3.68	*F* = 4.25**
	PR	≤ JH: 3.58	HS: 3.68	BA: 3.76	MA+: 3.88	*F* = 5.38**
	LS	≤ JH: 4.95	HS: 5.04	BA: 5.12	MA+: 5.24	*F* = 3.32*

PGI, personal growth initiative; PR, psychological resilience; LS, life satisfaction.

*p <0.05.

**p < 0.01.

### 3.6 Latent growth models

**FIGURE 2:**
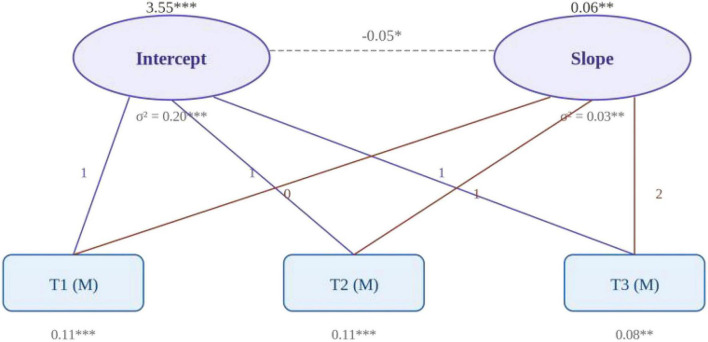
Latent growth model for personal growth initiative. *p < 0.05, **p < 0.01, ***p < 0.001.

**FIGURE 3:**
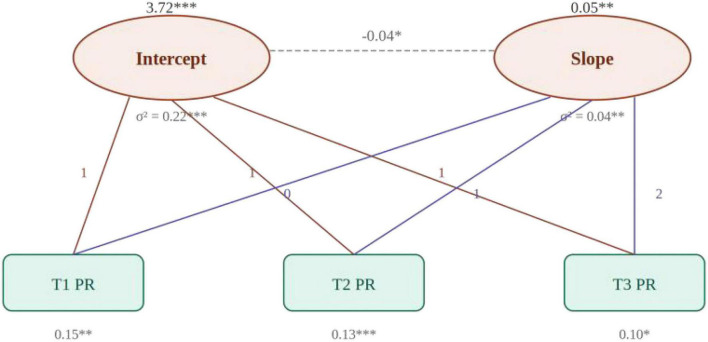
Latent growth model for psychological resilience. *p < 0.05, **p < 0.01, ***p < 0.001

**FIGURE 4:**
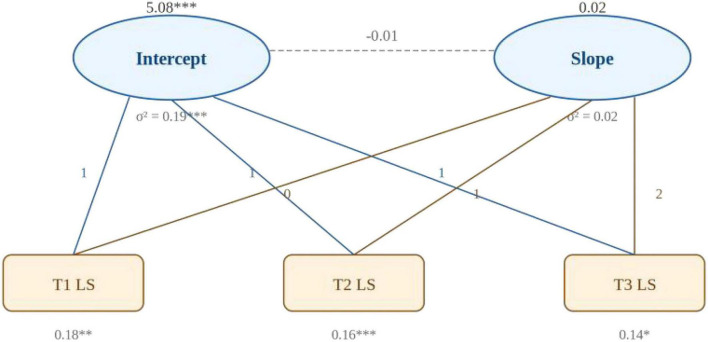
Latent growth model for life satisfaction. *p < 0.05, **p < 0.01, ***p < 0.001

Latent growth models were estimated to examine the developmental trajectories of personal growth initiative (Figure 2), psychological resilience (Figure 3), and life satisfaction (Figure 4). Model fit was acceptable for all three constructs (Table 6). Personal growth initiative showed a significant positive linear slope (slope = 0.06, *p* < 0.01), and psychological resilience also showed a significant positive linear slope (slope = 0.05, *p* < 0.01). In contrast, the slope of life satisfaction was not significant (slope = 0.02, *p* > 0.05), indicating relative stability over the 6-month period. These findings supported H3. For both personal growth initiative and psychological resilience, the covariance between intercept and slope was negative and significant, indicating slower growth rates for participants with higher initial levels.

**TABLE 6:** Latent growth model results.

Construct	χ ^2^(df)	CFI	TLI	RMSEA	SRMR	Intercept	Slope	Int. Var.	Slope Var.
PGI	2.28 (1)	0.99	0.98	0.035	0.021	3.55***	0.06**	0.20***	0.03**
PR	2.15 (1)	0.98	0.97	0.038	0.022	3.72***	0.05**	0.22***	0.04**
LS	1.86 (1)	0.99	0.98	0.028	0.019	5.08***	0.02	0.19***	0.02

PGI, personal growth initiative; PR, psychological resilience; LS, life satisfaction.

**p < 0.01.

***p < 0.001.

### 3.7 Cross-lagged and random-intercept cross-lagged models

**FIGURE 5:**
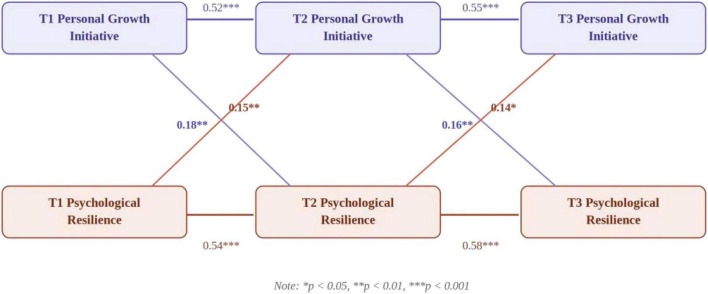
Cross-lagged panel model of personal growth initiative and psychological resilience. *p < 0.05, **p < 0.01, ***p < 0.001

The traditional cross-lagged panel model showed good fit (Figure 5): χ^2^(8) = 18.56, CFI = 0.98, TLI = 0.96, RMSEA = 0.044, SRMR = 0.032. Autoregressive effects were substantial for both personal growth initiative (β = 0.52–0.55, *p* < 0.001) and psychological resilience (β = 0.54–0.58, *p* <0.001). After controlling for these autoregressive effects, the reciprocal cross-lagged paths between personal growth initiative and psychological resilience were positive and significant in the traditional model, with standardized coefficients ranging from 0.14 to 0.18 (*p* <0.05; Table 7). These effect sizes are classified as small-to-medium per Cohen (1988).

**TABLE 7:** Summary of cross-lagged and RI-CLPM results.

Model	Path	β	*p*	Interpretation
CLPM	PGI → PR, adjacent	0.14–0.18	<0.05	Positive reciprocal
CLPM	PR → PGI, adjacent	0.14–0.18	<0.05	Positive reciprocal
RI-CLPM	T1 PGI → T2 PR	0.11	<0.05	Within-person retained
RI-CLPM	T1 PR → T2 PGI	0.09	<0.05	Within-person retained
RI-CLPM	T2 PGI → T3 PR	0.08	0.07	Marginal
RI-CLPM	T2 PR → T3 PGI	0.07	0.09	Marginal

PGI, personal growth initiative; PR, psychological resilience. RI-CLPM, random-intercept cross-lagged panel model.

To address the limitation that traditional cross-lagged panel models do not separate within-person and between-person effects, supplementary random-intercept cross-lagged panel models were estimated. The RI-CLPM showed acceptable fit: χ^2^(4) = 12.34, CFI = 0.97, TLI = 0.94, RMSEA = 0.055, SRMR = 0.035. Random intercept variances were significant for both personal growth initiative (σ^2^ = 0.28, *p* < 0.001) and psychological resilience (σ^2^ = 0.32, *p* < 0.001), indicating substantial stable between-person differences. The random intercepts were positively correlated (*r* = 0.52, *p* < 0.001). At the within-person level, the T1-to-T2 reciprocal paths remained significant: T1 personal growth initiative predicted T2 psychological resilience (β = 0.11, *p* < 0.05), and T1 psychological resilience predicted T2 personal growth initiative (β = 0.09, *p* < 0.05). The T2-to-T3 within-person paths were in the expected direction but only marginally significant (β = 0.08, *p* = 0.07 and β = 0.07, *p* = 0.09, respectively). Thus, the reciprocal association should be interpreted as modest and partially supported rather than uniformly robust across all adjacent waves. These findings provided qualified support for H4.

### 3.8 Longitudinal mediation

**FIGURE 6:**
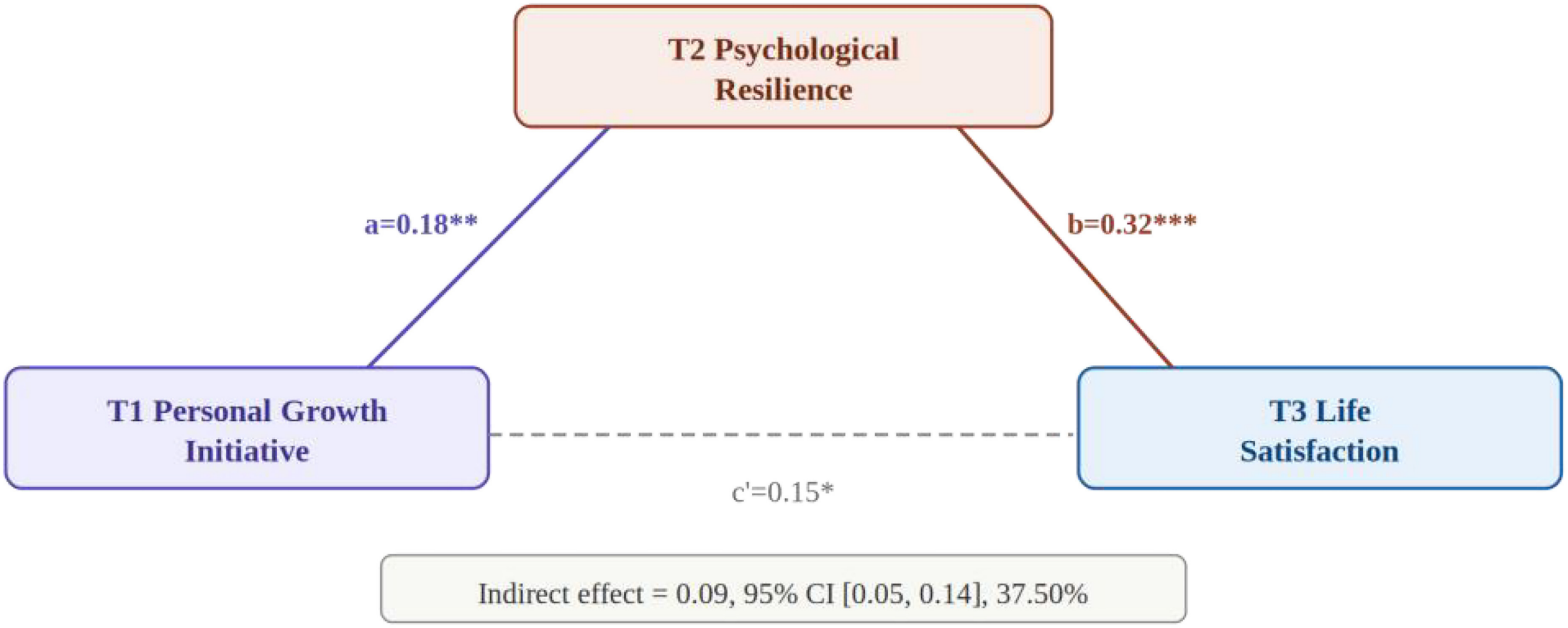
Longitudinal mediation model: mediating role of psychological resilience. *p < 0.05, **p < 0.01, ***p < 0.001

A longitudinal mediation model was tested with T1 personal growth initiative as the independent variable, T2 psychological resilience as the mediator, and T3 life satisfaction as the dependent variable (Figure 6). The model controlled for T1 psychological resilience, T1 life satisfaction, gender, age, education, marital status, number of children, and socioeconomic status. Model fit was good (Table 8): χ^2^(6) = 14.28, CFI = 0.98, TLI = 0.97, RMSEA = 0.045, SRMR = 0.028. The indirect effect through T2 psychological resilience was significant [indirect effect = 0.09, 95% CI (0.05, 0.14)], accounting for 37.50% of the total effect. According to Preacher and Kelley’s (2011) criteria, this proportion falls within the medium range. These results supported H5, while also indicating that the pathway should be interpreted as longitudinal association rather than causal proof.

**TABLE 8:** Longitudinal mediation model.

Path/effect	Estimate	95% CI	*p*/Note
T1 PGI → T2 PR	0.18	–	<0.01
T2 PR → T3 LS	0.32	–	<0.001
T1 PGI → T3 LS (direct)	0.15	–	<0.05
Indirect via T2 PR	0.09	[0.05, 0.14]	Significant
Proportion mediated	37.50%	–	Partial mediation

PGI, personal growth initiative; PR, psychological resilience; LS, life satisfaction. The model controlled for T1 PR, T1 LS, and demographic covariates.

## 4 Discussion

This study examined the developmental patterns and interrelations of personal growth initiative, psychological resilience, and life satisfaction among parents and primary caregivers of preschool children in the context of home-kindergarten co-education. The findings generally supported the proposed context–motivation–resources–outcomes framework. At baseline, the three psychological resources were positively associated. Psychological resilience statistically mediated the association between personal growth initiative and life satisfaction. Over 6 months, personal growth initiative and psychological resilience increased, whereas life satisfaction remained relatively stable. Longitudinal analyses further suggested modest reciprocal associations between personal growth initiative and psychological resilience and a longitudinal mediation pathway from T1 personal growth initiative to T3 life satisfaction through T2 psychological resilience.

These findings should be interpreted cautiously. The study used a longitudinal design and controlled for several baseline variables, but it remains observational. Therefore, the results are consistent with the proposed theoretical pathway but do not establish causality. Unmeasured factors such as partner relationship quality, kindergarten climate, teacher-family communication quality, major life events, and personality traits may also contribute to the observed associations.

### 4.1 Associations and mediating pathways

The positive associations among personal growth initiative, psychological resilience, and life satisfaction are consistent with prior research on proactive personality resources, adaptive coping, and subjective wellbeing (Hu et al., 2015; Robitschek and Keyes, 2009; Sun et al., 2014). The cross-sectional mediation result suggests that adults with higher personal growth initiative may also report higher resilience, which in turn is associated with higher life satisfaction. This pattern is consistent with conservation of resources theory, which proposes that resource investment and resource accumulation are linked to wellbeing outcomes (Hobfoll, 2001).

The cross-sectional mediating proportion of 38.30% is comparable to the 35.6% reported by García et al. (2023) in their study of Southeast Asian American parents of preschool children. The slightly larger proportion in the present study may reflect the structured nature of China’s home-kindergarten co-education policies, which provide systematic institutional channels for parental engagement that may amplify the pathway from growth motivation to resilience accumulation. From the perspective of parental involvement theory (Hoover-Dempsey and Sandler, 1997), parents and caregivers with higher growth initiative may perceive stronger role obligations for educational participation, develop greater self-efficacy, and consequently invest more consistently in their parenting resources.

The longitudinal mediation result extends this pattern by introducing temporal ordering. T1 personal growth initiative was associated with T3 life satisfaction through T2 psychological resilience (indirect effect = 0.09, 37.50% of total effect). This proportion falls within the medium range according to Preacher and Kelley’s (2011) criteria, suggesting that resilience may serve as a substantively important bridge variable rather than a trivial indirect route. However, the mediation result should be treated as a statistical pathway rather than proof of a causal chain. Future intervention studies are needed to test whether increasing personal growth initiative or resilience can produce subsequent changes in life satisfaction.

### 4.2 Developmental trajectories and reciprocal associations

The latent growth models showed that personal growth initiative and psychological resilience increased over 6 months. This finding is consistent with conservation of resources theory’s resource gain spiral hypothesis (Hobfoll, 2001) and with the view that motivational and adaptive resources can develop through continued participation in family-kindergarten interactions. Home-kindergarten co-education may provide opportunities for adults to communicate with teachers, observe other families, access parenting information, and practice coping strategies. The ceiling effect observed—indicated by the negative intercept-slope covariance—suggests that participants with higher initial levels showed slower growth, which has practical implications for targeting intervention efforts toward those with lower baseline resources.

Life satisfaction, in contrast, remained relatively stable. This is consistent with adaptation theories of subjective wellbeing (Brickman and Campbell, 1971; Lucas, 2007), which suggest that life satisfaction may change more slowly than specific psychological resources. Practically, this means that interventions should not expect rapid changes in life satisfaction. Personal growth initiative and resilience may be more sensitive short-term indicators, whereas life satisfaction may be more appropriate as a longer-term outcome.

The traditional cross-lagged model suggested reciprocal associations between personal growth initiative and psychological resilience. Supplementary RI-CLPM analyses provided partial support for this pattern at the within-person level. The significant T1-to-T2 paths suggest that when individuals reported higher-than-usual personal growth initiative, they tended to report higher-than-usual resilience at the next wave, and vice versa. However, the T2-to-T3 within-person paths were only marginally significant. This weaker later-wave pattern may reflect estimation constraints inherent to RI-CLPM with only three waves (Berry and Willoughby, 2017), but it also cautions against overstating the robustness of the reciprocal process. Compared to Smith et al.’s (2022) findings on reciprocal effects of parental sensitivity and resilience, the present study provides complementary evidence in the Chinese cultural context, though with a different variable pair.

### 4.3 Demographic differences and differentiated support

The demographic findings point to the need for differentiated parent and caregiver support. Female participants reported higher personal growth initiative and psychological resilience than male participants, which is consistent with international research showing that mothers typically demonstrate greater engagement in children’s education (Fantuzzo et al., 2004; McWayne et al., 2004). From the perspective of parental involvement theory (Hoover-Dempsey and Sandler, 1997), this gender gap may reflect differences in role construction—the extent to which adults see active educational engagement as part of their caregiving identity. This finding suggests that home-kindergarten programs should more explicitly invite and support fathers’ participation rather than assuming mothers as the default participants.

Participants aged 36–45 showed relatively high psychological resilience, whereas older caregivers aged 46–55 showed meaningful growth potential in personal growth initiative over time. Viewed through a life-span developmental lens (Baltes et al., 2006), the older group’s lower initial growth initiative may reflect the dual burden of career-stage demands and renewed parenting responsibilities rather than a lack of capacity for growth. Their significant growth trajectory suggests that with appropriate support, this group can develop comparable psychological resources. Kindergartens may therefore need to provide more accessible communication formats and practical guidance for older caregivers.

Educational differences were also evident. Participants with higher educational attainment reported stronger psychological resources. This pattern raises an equity concern: adults with lower educational attainment may have fewer resources but greater need for support. Practical interventions should use clear language, concrete examples, peer mentoring, and community-based workshops to avoid excluding families with lower educational resources.

### 4.4 Theoretical contributions

The study contributes to the literature in three ways. First, it brings together personal growth initiative, psychological resilience, and life satisfaction within the context of home-kindergarten co-education. This helps shift the focus from behavioral participation alone to the psychological resources that may sustain participation, complementing Epstein’s (2019) framework, which emphasizes that effective family-school partnerships require not only institutional structures but also internal psychological readiness. Second, the study uses longitudinal data to examine both developmental trajectories and temporal pathways, providing stronger evidence than cross-sectional designs alone. Third, by supplementing traditional CLPM with RI-CLPM, the study acknowledges an important methodological debate and offers a more cautious interpretation of reciprocal associations.

### 4.5 Practical implications

The findings suggest that home-kindergarten co-education programs should include psychological support for parents and primary caregivers. Kindergartens can support personal growth initiative by providing accessible parenting resources, encouraging reflective participation, and recognizing caregivers as active partners. They can support resilience by offering peer support groups, stress-management workshops, teacher consultations, and mentoring between experienced and newer caregivers. Because the association between personal growth initiative and resilience appears reciprocal, support programs should be designed as cyclical rather than one-time activities, with ongoing opportunities for both growth initiative activation and resilience reinforcement.

Differentiated support is also needed. Fathers may require targeted invitations and role-oriented activities. Older caregivers may benefit from digital literacy support and practical communication assistance. Caregivers with lower educational attainment may benefit from simplified materials and peer-based guidance. These approaches may help home-kindergarten co-education move beyond formal participation toward more equitable and psychologically supportive partnerships.

### 4.6 Limitations and future directions

Several limitations should be acknowledged. First, the study used convenience sampling from eight kindergartens in five cities across three provinces. The findings may not generalize to rural regions, western China, ethnic minority communities, or other cultural contexts. Urban-rural differences in home-kindergarten co-education infrastructure and parenting norms may produce different patterns that the current sample cannot capture. Future studies should use stratified random sampling and broader regional coverage.

Second, the 6-month tracking period and three-wave design limited the ability to examine nonlinear trajectories and stable within-person dynamics. The RI-CLPM’s marginal T2-to-T3 effects may partly reflect estimation constraints with only three waves. Future studies should extend the follow-up period to 1 or 2 years and include four to six measurement waves to allow more robust RI-CLPM estimation and more precise tests of developmental change.

Third, all main variables were measured through self-report questionnaires. Although common method bias tests did not indicate serious bias, future studies should include teacher reports, kindergarten participation records, behavioral observations, or digital communication data. Multi-informant and multi-method designs would strengthen the evidence.

Fourth, this study treated home-kindergarten co-education primarily as the policy and practice context rather than as an explicitly measured construct. Future research should directly assess home-kindergarten communication quality, perceived teacher support, parental involvement frequency, and institutional resources. Including these variables would allow stronger tests of how co-education conditions shape psychological resources.

Fifth, although several demographic and socioeconomic covariates were controlled, other potential confounders—such as partner relationship quality, kindergarten quality, teacher-child relationship quality, personality traits, and major life events—were not fully assessed. Finally, because the study was observational, causal claims cannot be made. Randomized intervention studies are needed to test whether enhancing personal growth initiative or resilience can improve caregiver wellbeing and home-kindergarten collaboration.

## 5 Conclusion

This study examined personal growth initiative, psychological resilience, and life satisfaction among parents and primary caregivers of preschool children in the context of home-kindergarten co-education. The results showed that the three psychological resources were positively associated at baseline and that psychological resilience partially mediated the association between personal growth initiative and life satisfaction. Longitudinally, personal growth initiative and psychological resilience increased over 6 months, whereas life satisfaction remained relatively stable. Personal growth initiative and psychological resilience showed modest reciprocal associations, and T2 psychological resilience mediated the association between T1 personal growth initiative and T3 life satisfaction.

Overall, the findings suggest that psychological resilience may be an important bridge linking proactive growth motivation with wellbeing among adults involved in preschool children’s education. Home-kindergarten co-education programs may benefit from moving beyond participation arrangements alone and incorporating differentiated psychological support for parents and primary caregivers.

## Data Availability

The original contributions presented in this study are included in the article/supplementary material, further inquiries can be directed to the corresponding author.
